# PCNL in neurogenic bladder: A challenging population for both clinical management and analysis

**DOI:** 10.1002/bco2.268

**Published:** 2023-06-30

**Authors:** Bridget Heijkoop, Bodie Chislett, Marlon Perera, Stephen Esler, Damien Bolton, David Rowan Webb

**Affiliations:** ^1^ Austin Hospital Heidelberg Victoria Australia; ^2^ Department of Urology Austin Health Heidelberg Victoria Australia; ^3^ Department of Surgery University of Melbourne Melbourne Victoria Australia

**Keywords:** neurogenic bladder, PCNL, percutaneous nephrolithotomy, renal stones, spinal cord injury

## Abstract

**Objectives:**

To review the management of patients with neurogenic bladder undergoing percutaneous nephrolithotomy (PCNL) at our institution with the aim of assessing peri‐operative morbidity.

**Subjects/patients and methods:**

We conducted a retrospective review of all neurogenic bladder patients who underwent PCNL at our hospital in the last decade with the aim of assessing peri‐operative morbidity.

**Results:**

A total of 298 PCNL were performed during the study period of which 58 were in patients with a neurogenic bladder or urinary diversion, 33 of which were in SCI patients. Preoperative demographic and stone characteristics, intraoperative data and postoperative length of stay and complications are summarised in table form.

**Conclusion:**

PCNL remains an acceptably safe and efficacious treatment for upper tract stone disease in patients with neurogenic bladders and will continue to have a valuable role where SCI prevents alternative approaches such as ureteroscopy.

## 1 INTRODUCTION

Neurogenic bladder secondary to spinal cord injury (SCI) or other pathologies such as multiple sclerosis (MS), spina bifida (SB) or cerebral palsy (CP) is a major risk factor for renal stones. SCI patients are estimated to have a 7%–20% risk of developing stones over a 10‐year period^1^, ^2^, ^3^ and lifetime risk up to six times that of the general population.^3^ Contributing factors include neurogenic bladder with or without vesicoureteric reflux, recurrent and chronic bacteriuria and bladder management techniques including indwelling or intermittent catheterisation, and immobility causing bone demineralisation and hypercalciuria, especially in the immediate post‐injury period.^4^, ^5^ Individual patient characteristics vary widely dependent on the causative pathology, timeframe of disease state and other associated conditions.

Stone disease in these patients is frequently further complicated by urinary tract infection (UTI) and urosepsis, due to recurrent or chronic bacteriuria from catheterisation. This is significant as sepsis is the most common cause of mortality in patients with kidney stone disease.^6^ Finally, altered sensory and motor function can result in atypical presentations of renal colic or urosepsis such as with autonomic dysreflexia^2^, ^5^ or hyperhidrosis,^7^ making diagnosis and management challenging.

Elective percutaneous nephrolithotomy (PCNL) is a well‐established approach in treatment of upper tract renal stone disease.^5^, ^6^ The majority are conducted as a single‐stage operation with percutaneous flank access to the kidney performed after obtaining retrograde ureteric access. Choice of instruments and patient positioning (prone or supine) are usually determined by surgeon preference rather than specific clinical features or any strong evidence base for superiority of one approach. Successful PCNL relies on selecting the most appropriate approach to the individual stone. In particular, this is dependent on skin to stone distance. Retrograde access to the ureter is important to facilitate contrast injection to the collecting system and intraoperative imaging.

Advantages of PCNL in the neurogenic bladder patient include the ability to clear a large stone burden in a single procedure and access stones where treatments such as ureteroscopy are impossible due to disease state, for example, limb contractures preventing adequate lithotomy positioning. However, abnormalities of anatomy or physiology in this population can increase complexity of PCNL or necessitate a particular position or approach. In particular, barriers to optimal position can include body wall distortion, resulting in the kidney being located almost entirely in the rib cage or abnormally related to other intra‐abdominal organs, limb contractures precluding retrograde access or prone positioning. Patients may also be at risk of secondary neurological injury and have compromised respiratory function and altered sensory and motor function increasing risk of intraoperative, anaesthetic and postoperative conditions such as autonomic dysreflexia and pressure injuries or causing delay in identification of complications postoperatively.

Our aim is to review the management of patients with neurogenic bladder undergoing PCNL at our institution with the aim of assessing peri‐operative morbidity.

### 1.1 Subjects/patients and methods

We retrospectively reviewed data from all adult (>18 years) patients undergoing PCNL at our hospital from 1 January 2010 to 31 December 2021 inclusive. Cases were included in the analysis if they were documented to have an existing condition causing neurogenic bladder at the time of PCNL. Cases were excluded if they were abandoned prior to gaining access to the kidney.

At our institution, all PCNL patients undergo preoperative urine culture testing with antibiotic regime determined by culture results; any preoperative infection is treated according to sensitivities prior to proceeding with PCNL, and patients with negative cultures receive prophylactic ampicillin/gentamicin at induction, modified to allow for allergies and renal function on an individualised basis. Puncture of the kidney is primarily performed by the operating urologist under fluoroscopic X‐ray guidance; however, in select patients where a nephrostomy tube previously inserted by the radiology department (e.g. for decompression in the setting of sepsis) is present, this may be utilised by the urologist to access the collecting system if suitably positioned for tract dilation and adequate stone access. Supine versus prone positioning of the patient is determined by the surgeon on a case by case assessment taking into account patient and stone characteristics as well as surgeon experience and preference with each approach. Potentially difficult cases are presented preoperatively in a multidisciplinary team meeting attended by urology, radiology and pathology departments.

Outcome measures included operative time, number of punctures, postoperative length of stay (LOS), ICU admission and length or stay, complications and subsequent episodes of stone disease.

Subgroup analysis by causative pathology was initially planned to enable comparison by causative pathology and urinary system characteristics; however, due to limited population size precluding any statistically significant analysis, subgroup analysis was performed only on the SCI group.

Endpoints were grouped into demographic, preoperative and access, intraoperative and postoperative and complication data. Demographic data included patient age, gender and Charlson comorbidity score. Preoperative and access data included laterality of the target kidney, abnormalities of kidney or body wall anatomy, stone size and location, preoperative use of antibiotics to treat UTI, patient positioning and use of a preoperatively placed nephrostomy for renal access. Intraoperative data included operative time, type of retrograde access to the kidney, number of puncture attempts, sheath size and type of drainage (nephrostomy, ureteric stent or both) at completion of PCNL. Postoperative and complication data included mortality, complications graded by Clavien Dindo score, ICU admission and LOS, overall LOS and known subsequent episodes of stone disease.

## 2 RESULTS

A total of 298 PCNL were performed at our hospital during the study period. Of these, 58 (19%) were in patients with a neurogenic bladder or urinary diversion. A further six patients were excluded due to the indication for their urinary diversion being for reasons other than a neurogenic bladder (e.g. post‐cystoprostatectomy for malignancy) due to the absence of other systemic features of disease such as altered mobility, sensation or compromised respiratory function in these patients potentially confounding results, leaving 52 included patients. Thirty‐three of the 52 included patient (63%) were in SCI patients; however, four SCI patients had also undergone urinary diversion with an ileal conduit for bladder management. These four patients were included in overall analysis (52 patients with any form of neurogenic bladder or urinary diversion for neurogenic bladder), but excluded from the SCI subpopulation analysis to ensure results in the SCI group reflected changes in bladder function due to SCI rather than the conduit, leaving 29 included patients in the SCI subgroup.

### 2.1 Results: Demographic data

Patient age ranged in the overall cohort between 20 and 83 years with a mean of 45 years and median of 47 years. Causative pathology in included cases is summarised by Table 1.

**TABLE 1:** Causative pathology of neurogenic bladder and stone characteristics.

Causative pathology	Number of patients	
SCI with anatomical bladder	29	
SCI with ileal conduit	4	
Multiple sclerosis	4	
Spina bifida with anatomical bladder	1	
Spina bifida with ileal conduit	7	
Cerebral palsy	4	
Spinal cord regression	3	

Stone characteristics (number of patients with this stone characteristic)		
	Neurogenic bladder (any pathology)	SCI population
Staghorn (complete)	15	11
Partial staghorn or stone in multiple calyces	17	9
Upper pole	3	3
Lower pole	8	3
Pelvis/PUJ/ureteric	9	3

In the SCI group, patient age range was between 20 and 83 with a mean of 43 and median of 40. Twenty‐eight (97%) were in male patients and one (3%) in a female. Patient's preoperative Charlson comorbidity score ranged between two and nine with a mean of 2.9 and median of 2.^8^ Level of SCI was between C1 and T8 with 25 PCNL performed in patients with cervical level SCI and four in those with thoracic SCI. SCI was recorded to be incomplete in only three cases, all of which were thoracic level SCI. Bladder management was with SPC in 23 cases, intermittent clean catheterisation in five cases and long‐term urethral catheter in one case.

### 2.2 Results: Stone characteristics

For the entire cohort, estimated total preoperative stone burden was greater than 2 cm in 44 (84%). Stone analysis was available in 14 cases, including calcium oxalate, calcium phosphate and struvite stones. Location of the stone within the collecting system is summarised in Table 1.

In the SCI cohort, preoperative stone size was available for 27 of the 29 included patients, with 26 greater than 2 cm diameter. Stone analysis included struvite, calcium oxalate, calcium phosphate and mixed composition.

### 2.3 Results: Preoperative and access data

In the SCI group, three cases (10.3%) had additional abnormalities of renal anatomy, including two duplex systems (stone located in the lower moiety in both duplex cases), and one stone was partially located in a calyceal diverticulum (upper pole). Twelve cases (41.4%) had had a previous PCNL. Positioning was prone in 18 (62%) and supine in 11 (38%). Thirteen (45%) cases had a pre‐existing nephrostomy tube in place although this was not always used for the PCNL access tract.

### 2.4 Results: Intraoperative characteristics

Intraoperative characteristics of completed PCNL cases are summarised in Table 2.

**TABLE 2:** Intraoperative characteristics.

Intraoperative characteristics (Result reported separately for entire neurogenic bladder population (All) and for SCI subgroup (SCI))				
Operative time (minutes)	Minimum	Maximum	Mean	Median
	54 (All) 60 (SCI)	226 (All) 225 (SCI)	131 (All) 140 (SCI)	121.5 (All) 130 (SCI)
Type of retrograde access	None	Ureteric catheter	Access sheath	Concurrent ureteroscopy
	25 (All) 9 (SCI)	25 (All) 18 (SCI)	1 (All) 1 (SCI)	1 (All) 1 (SCI)
Punctures	1		>1	
	45 (All) 25 (SCI)		7 (All) 4 (SCI)	
Sheath size	Nephrostomy only or failed		Mini (16–22 Fr)	Standard (24–48 Fr)
	4 (All) 2 (SCI)		17 (All) 6 (SCI)	31 (All) 21 (SCI)
Postoperative drainage (one nil as abandoned prior to access)	Nephrostomy and ureteric stent		Nephrostomy	Ureteric stent only
	7 (All) 4 (SCI)		41 (All) 23 (SCI)	3 (All) 2 (SCI)

Eight cases (15%) were not included in the analysis in Table 3 due to being abandoned prior to any stone destruction or removal, including four failed access to the collecting system and three cases where pus was aspirated on accessing the kidney and one case where bowel injury was identified on dilation of the initial tract. Four of these were from the SCI group (14% of SCI cases), two in SB patients both with an ileal conduit, one in a CB patient and one in a spinal cord regression patient. Of the four cases where the reason for abandoning PCNL was failed access, one was converted to a flexible ureteroscopy and laser, one had a subsequent successful attempt at PCNL, and one was referred for extracorporeal shock wave lithotripsy (ESWL) having also previously failed an attempt at ureteroscopic access, and one died due to respiratory complications prior to further attempts at stone clearance. Preoperative Charlson comorbidity score in the abandoned patients ranged between one and eight. Using the CLASSIC classification of intraoperative complications, with the exception of one patient death (grade 4) and bowel injury as a grade 3, the remainder were grade 2.^9^

**TABLE 3:** Postoperative complications and Clavien Dindo grade.

Complications						
	Uncomplicated UTI (Clavien Dindo 2)	Urosepsis (Req ICU/additional procedure) (Clavien Dindo 4a)	Bleeding at nephrostomy (managed on ward) (Clavien Dindo 1)	Anaemia requiring transfusion (Clavien Dindo 2)	Pseudoaneurysm (required embolisation) (Clavien Dindo 3a)	Autonomic dysreflexia (Clavien Dindo 2)
Total cohort	6 (13.6%)	7 (15.9%)	1 (2.3%)	3 (6.8%)	1 (2.3%)	3 (5.17%)
SCI population	5 (20%)	5 (20%)	1 (4%)	1 (4%)	1 (4%)	3 (12%)

*Note*: % scores calculated from cases proceeding with stone destruction/removal; 52 total cases, 25 SCI cases.

### 2.5 Results: Postoperative and complications

Cases abandoned prior to any stone destruction or extraction were excluded from postoperative complication analysis.

In those cases where PCNL was completed, postoperative LOS ranged from two to 23 days in both groups with a mean and median of 6.61 and 4.5 days in the overall population and 7 and 5 days in the SCI population. Seven patients required ICU admission, two of which were planned ICU admissions and five of which were SCI patients. Both planned ICU admissions were in non‐SCI patients. ICU LOS was between 1 and 8 days in both groups with a mean and median of 3.14 and 4 days in the overall population and 5 and 6 days in the SCI group.

Postoperative complications are summarised in Table 3. Clavien Dindo grade of complications ranged between one and four with a mean and median of 2.5 and 2 in both the overall and SCI populations.^10^ All patients who developed urosepsis postoperatively had received appropriate preoperative treatment of UTI in addition to antibiotics at induction.

At completion of the procedure, 20 (45%) patients were considered cleared of stone; however, 27 (61%) had a subsequent episode of stone disease following the study procedure. In the SCI subgroup, 14 (48%) patients were considered cleared with 20 (69%) having a subsequent episode.

## 3 DISCUSSION

Recommendations on the use of PCNL specific to patients with significant abnormalities of urinary tract or body wall anatomy and or physiology are currently lacking. We hypothesise that this is due to the wide variety of causative pathologies and spectrum of severity within each, combined with the challenges of conducting research specifically in populations that represent a small proportion of the general population, resulting in a limited body of evidence being able to be generated.

Pre‐existing literature is also limited; to date, we have been unable to identify any existing prospective or randomised trials regarding PCNL specifically in the neurogenic bladder population, and there are few existing studies with patient cohorts of similar size or larger than our own. Existing studies are limited by heterogeneity within their population, with inclusion of varying causative pathologies and SCI of different levels resulting in inclusion of patients with vastly different bladder pathology within a single cohort for analysis.^11^, ^12^, ^13^, ^14^, ^15^ Even papers utilising large populations generated by retrospective multi‐institutional review such as that by Baldea et al. draw limited conclusions beyond confirming that PCNL in these patients is more challenging and less efficacious and carries higher morbidity and mortality than in the general population.^16^

These challenges in researching PCNL specifically within the neurogenic population are likely to persist, particularly as advances in ureteroscopy such as the continued refinement of instruments result in a declining overall number of PCNLs performed demonstrated by trends reported from MBS data.^17^ In particular, generating sufficient numbers of SCI patients to perform an appropriately powered comparison with a population with comparable bladder characteristics is likely to require a prohibitively lengthy period of data collection without coordinated participation from multiple institutions, which in itself can result in additional challenges.

Our study analyses over a decade's worth of data from a large tertiary centre that also functions as the state‐wide referral centre for SCI patients in a populous state. Despite this, population remained small (52 patients), and consequently, while our original aim was to compare our experience of PCNL in the neurogenic bladder population to that in patients with normal bladder physiology, an analysis of statistical significance was not possible for successful stone clearance or assessment of specific risk factors for complications. Male patients are also disproportionately represented, likely reflecting the predominance of male patients in the SCI population who comprise the majority of our total cohort.

Our current paper differs from the pre‐existing literature by the inclusion a subgroup analysis of exclusively SCI patients retaining their native bladder, improving on the earlier research by its reduced heterogeneity. All patients in this subgroup also had an injury level at T8 or higher and bladder management with a catheter (SPC, urethral or intermittent), meaning all would be expected to have similar bladder characteristics of a hypertonic detrusor and hypertonic urethral sphincter resulting in a high‐pressure bladder, mitigated by the presence of a catheter. Thus, while accepting the limitations of analysis in a cohort of this size, any conclusions drawn could be more confidently applied to other patients with supra‐sacral SCI.

Limitations of our own study include the inability to compare outcomes of our included population of neurogenic bladder patients undergoing PCNL with a matched population of neurogenic bladder patients with stones managed expectantly without intervention over the same timeframe. This was not possible due to initial identification of PCNL cases being obtained from theatre procedural coding information, and hence not encompassing any non‐operative patients. It was also outside the scope of this project to directly compare the outcomes of the neurogenic bladder cohort to a matched group of patients without neurogenic bladder undergoing PCNL from our own institution and thus results were compared to reported outcomes in the existing literature.

However, we believe there is still considerable value in sharing experience and strategies in the management of these most difficult PCNL patients. Our results suggest PCNL continues to be an acceptably safe intervention for patients with significant renal stone burden and concurrent neurogenic bladder, providing an individualised approach to the stone and patient is taken. We observed a moderate overall complication rate, as well as low rates of ICU admission and major complications (defined as those scored as Clavien Dindo 3 or above), although there was discrepancy between the estimated stone clearance rate and observed rate of recurrent stone disease. Our results regarding safety are comparable to rates published in the existing literature where mortality of up to 4.2% and major complication rates between 6% and 25.7% are reported, although stone clearance rate was lower than that reported for SCI patients undergoing PCNL in previous papers bu Lawrentschuk et al. (87% in 54 PCNL), Knox et al. (69.7% in 47 patients) and Culkin et al. (95.7% in 35 SCI patients).^11^, ^12^, ^13^, ^14^ The high number of cases performed without retrograde access to the kidney in our study (25/44, 57% overall and 9/29, 31% in the SCI cohort) demonstrates the continued need for PCNL in this cohort where alternative modalities of stone treatment such as ureteroscopy may not be possible due to sequelae of the SCI or associated conditions such as limb contractures.

For neurogenic bladder patients requiring PCNL in future, we recommend that treatment is individualised to the patient's own anatomical and stone characteristics, but at a minimum, the following conditions should be met:

- Preoperative up‐to‐date computed tomography (CT) imaging obtained to enable accurate assessment of skin to stone distance, optimal calyx for puncture and relationship of planned access tract to neighbouring organs and structures at risk.
- Retrograde access to the collecting system planned, with alternative strategies made in the event this is unsuccessful.
- Preoperative urine MCS taken with any infection treated, and the procedure should not proceed if any evidence of inadequately treated infection is encountered.
- Consideration of individual patient characteristics requiring modifications such as specific pressure are padding and additional respiratory supports.
- Assumption made that the condition resulting in a neurogenic bladder also increases the patient's risk for intraoperative and postoperative complications and thus there should be a low threshold for planning HDU or ICU support postoperatively

We also benefit greatly from the assistance of interventional radiology department, with preoperative nephrostomy allowing accurate preoperative urine MCS, decompression of the upper tract and an alternative approach for contrast delivery, although the nephrostomy itself is rarely suitable for use as the working puncture due to its length, location and curvature.

Consideration of the development of a nationwide registry of SCI patients and/or other neurogenic bladder patients undergoing may also help to facilitate the generation of more powerful data to benefit additional research in these populations in future.

## 4 CONCLUSION

Neurogenic patients requiring PCNL for stone management present numerous challenges, both in clinical management and obtaining data of sufficient power to enable statistically significant analysis in this population to better guide treatment. Currently, existing data are limited by small, heterogeneous cohorts, but it appears PCNL remains acceptably safe and efficacious for patients with neurogenic bladders and will continue to have a valuable role where SCI prevents alternative approaches such as ureteroscopy accepting the rate of complete clearance likely to be lower than that in patients with normal bladder anatomy and physiology.

## AUTHOR CONTRIBUTIONS

**Bridget Heijkoop**: Conceptualisation, methodology, investigation, data curation, writing—original draft, writing—review and editing. **Bodie Chislett**: Investigation. **Marlon Perera**: Conceptualisation, writing—review and editing. **Stephen Esler**: Writing—review and editing. **Damien Bolton**: Conceptualisation, writing—review and editing, supervision. **David Rowan Webb**: Conceptualisation, writing—review and editing, supervision.

## CONFLICT OF INTEREST STATEMENT

None to declare.

## Data Availability

De‐identified data are available from the corresponding author on request.
